# Altered Spatiotemporal and Kinematic Gait in Patients with Knee Osteoarthritis

**DOI:** 10.3390/jfmk11020137

**Published:** 2026-03-26

**Authors:** Plaiwan Suttanon, Praewpun Saelee, Sudarat Apibantaweesakul

**Affiliations:** 1 Department of Physical Therapy, Faculty of Allied Health Sciences, Thammasat University, Pathum Thani 12121, Thailand; lee.praewpun@gmail.com; 2 Thammasat University Research Unit in Health, Physical Performance, Movement and Quality of Life for Longevity Society, Thammasat University, Pathum Thani 12121, Thailand; 3 Department of Sports Science and Sports Development, Faculty of Allied Health Sciences, Thammasat University, Pathum Thani 12121, Thailand

**Keywords:** knee osteoarthritis, gait kinematics, older adults, spatiotemporal analysis, 3D motion analysis

## Abstract

**Background:** Knee osteoarthritis (KOA) is a major cause of pain, mobility limitation, and increased fall risk among older adults. Gait dysfunction, characterized by spatiotemporal and kinematic alterations, is a key functional consequence of KOA. While sagittal-plane gait deviations are well-established, multiplanar kinematic changes—particularly in the frontal and transverse planes—remain less clearly understood. This study aimed to compare three-dimensional gait characteristics between older adults with and without KOA. **Methods:** Ninety older adults (45 with KOA and 45 controls) completed gait assessments using a VICON™ motion capture system. Participants walked at a self-selected speed along a straight walkway without turning movements during data collection. Spatiotemporal parameters and lower-limb joint kinematics (hip, knee, and ankle) were recorded during key gait phases: initial contact, mid-stance, toe-off, and mid-swing. Group comparisons were performed using independent *t*-tests with statistical significance set at *p* < 0.05. **Results:** Compared with controls, participants with KOA demonstrated significantly slower gait velocity (*p* = 0.001), reduced cadence (*p* = 0.020), shorter stride length (*p* = 0.011), increased step time (*p* = 0.006), prolonged double support time (*p* = 0.009), and reduced single support time (*p* = 0.012). Kinematic analysis revealed greater knee adduction at initial contact (*p* = 0.001), reduced hip adduction (*p* = 0.002) and greater knee adduction (*p* = 0.003) during mid-stance, and increased ankle plantarflexion at toe-off (*p* = 0.004) in the KOA group. No significant between-group differences were observed during the mid-swing phase. **Conclusions:** Older adults with KOA exhibit distinct spatiotemporal and multiplanar kinematic gait alterations, particularly during weight-bearing phases. These changes may reflect adaptive gait patterns associated with joint dysfunction rather than definitive compensatory mechanisms. Three-dimensional gait analysis may provide valuable biomechanical insights to support early identification of mobility impairments and inform targeted rehabilitation planning in individuals with KOA.

## 1. Introduction

Osteoarthritis is a leading cause of pain, disability, and mobility limitation in older adults worldwide [1,2]. According to global estimates, in 2019, over 528 million people were affected by osteoarthritis, with the knee joint being the most commonly involved site [3]. The prevalence of knee osteoarthritis (KOA) increases markedly with age, posing a substantial burden on functional independence and quality of life [1,2]. The disease is characterized by progressive cartilage degradation, joint space narrowing, and structural bone changes, often resulting in pain, stiffness, and functional impairments [4]. Gait dysfunction is a hallmark clinical manifestation of knee osteoarthritis (KOA) and is associated with biomechanical and neuromuscular alterations, including abnormal joint kinematics and kinetics, impaired muscle activation patterns, reduced proprioceptive acuity, and diminished motor control that collectively affect movement coordination and stability [5,6].

Mechanically, individuals with knee OA frequently adopt compensatory gait strategies, defined as adaptive movement modifications that arise in response to pain, joint instability, muscle weakness, and reduced joint range of motion. These strategies aim to reduce mechanical loading on the affected joint and maintain functional mobility, but may also alter walking efficiency and stability. Common compensatory patterns include reduced gait speed, shorter stride length, and increased double support time [7,8]. These spatiotemporal modifications may temporarily alleviate pain and improve balance control, but persistent compensatory behaviors can redistribute joint loading to adjacent joints and may compromise overall walking performance [9]. Altered gait mechanics are also closely linked to fall risk in older adults with KOA. Epidemiological studies show that older adults with symptomatic KOA have a 30–50% greater risk of falls, often during ambulation, compared to age-matched peers without osteoarthritis [10]. Falls may occur due to reduced dynamic stability, delayed neuromuscular responses, impaired proprioception, quadriceps weakness, and joint instability, which are frequently observed in KOA populations. Importantly, spatiotemporal gait characteristics such as slower walking speed, prolonged double support time, and shorter step and stride lengths have been associated with unstable gait patterns and fall susceptibility [11,12]. Compensatory strategies may therefore play a dual role: they can enhance short-term stability by increasing cautious gait patterns, yet may also reflect underlying neuromotor deficits that elevate fall risk. Consequently, identifying gait alterations associated with instability may facilitate early detection of individuals at elevated risk of falls.

Gait analysis using three-dimensional motion capture systems such as VICON offers detailed insights into kinematic patterns across all three planes of motion. While sagittal plane abnormalities are well-documented [6,8,9], evidence regarding multiplanar joint motion across different severity levels of KOA remains inconsistent. Previous studies suggest that frontal-plane deviations such as increased knee adduction angles may reflect altered medial–lateral load distribution, while transverse-plane rotational changes may influence tibiofemoral shear forces and cartilage stress; however, these mechanisms remain incompletely characterized, particularly during weight-bearing phases [6,13,14]. In addition to motion capture systems [5,6,7], several approaches have been used to assess gait characteristics in individuals with KOA. Clinical assessments, such as the 10-m walk test and Timed Up and Go test, provide practical measures of gait speed and functional mobility [12,15]. Wearable sensor-based technologies, including inertial measurement units (IMUs), enable the evaluation of spatiotemporal parameters in real environments [16,17]. Furthermore, force platforms are commonly used to quantify temporal gait characteristics and ground reaction forces [14]. However, these methods are limited in their ability to capture detailed multiplanar joint kinematics.

A clearer characterization of multiplanar gait alterations is important because abnormal transverse-plane rotational mechanics may alter tibiofemoral contact stresses and potentially contribute to structural disease progression, while frontal-plane deviations may compromise dynamic stability. Such multiplanar biomechanical changes are not fully captured by conventional sagittal-plane assessments, potentially limiting early identification of clinically relevant movement abnormalities. Furthermore, gait-based indicators have been proposed as potential biomechanical markers for identifying individuals at risk of disease progression or falls. However, the mechanisms linking KOA-related gait alterations to fall risk remain insufficiently clarified, and it is uncertain whether specific multiplanar kinematic deviations may serve as early indicators of instability.

Accordingly, this hypothesis-driven study aimed to compare three-dimensional gait kinematics between older adults with and without knee osteoarthritis using motion capture technology. Particular emphasis was placed on multiplanar joint kinematics of the hip, knee, and ankle during key weight-bearing phases of the gait cycle. We hypothesized that individuals with KOA would demonstrate greater multiplanar kinematic deviations compared with controls, particularly in frontal and transverse planes, reflecting compensatory adaptations associated with joint instability and altered load distribution.

By addressing underexplored multiplanar biomechanics and their potential clinical implications, this study may enhance biomechanical understanding of KOA-related gait adaptations and inform targeted rehabilitation strategies for improving mobility and safety in older adults.

## 2. Materials and Methods

### 2.1. Participants

A total of 90 community-dwelling older adults participated in this study, including 45 individuals clinically diagnosed with knee osteoarthritis (KOA) and 45 age-matched healthy controls without a history of knee pathology. Participants in the KOA group were diagnosed according to the American College of Rheumatology (ACR) clinical classification criteria. Clinical assessments were performed by a licensed physical therapist. Inclusion criteria were as follows: (1) age ≥ 60 years and (2) ability to ambulate independently without assistive devices for at least 10 m. Exclusion criteria were as follows: (1) history of other significant orthopedic conditions (e.g., recent lower limb surgery, joint replacement, or fracture within the past year), (2) intra-articular injection to the lower limb within the previous six months, and (3) presence of neurological, vestibular, or cardiovascular disorders that could impair gait or balance performance.

The study was approved by the Human Research Ethics Committee of Thammasat University (approval number: 085/2558). Written informed consent was obtained from all participants prior to enrollment.

### 2.2. Procedure

#### 2.2.1. Clinical and Demographic Assessments

Demographic data, including age, sex, height, weight, and body mass index (BMI), were collected. Participants also completed the modified 22-item Thai version of the Western Ontario and McMaster Universities Osteoarthritis Index (WOMAC) to assess self-reported joint pain (5 items), stiffness (2 items), and physical function (15 items). Each item was scored using a visual analog scale (VAS) from 0 to 9, with total subscale scores ranging from 0–45 (pain), 0–18 (stiffness), and 0–135 (function). Higher scores indicate greater symptom severity and functional impairment [18].

#### 2.2.2. Gait Data Collection

Three-dimensional kinematic data were acquired using a VICON™ motion capture system (Oxford Metrics Ltd., Oxford, UK) consisting of eight infrared cameras operating at a sampling frequency of 100 Hz. Prior to testing, participants changed into standardized form-fitting clothing and removed all accessories that could interfere with marker tracking.

**Figure 1:**
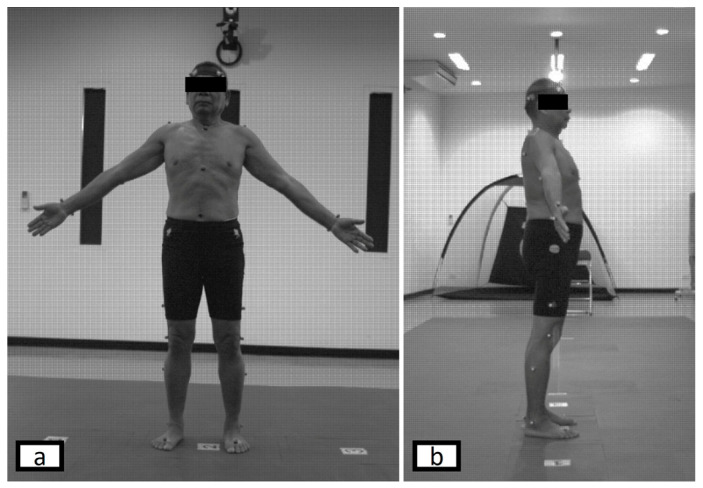
Positions of retroreflective markers: (**a**) anterior view and (**b**) lateral view.

**Figure 2:**
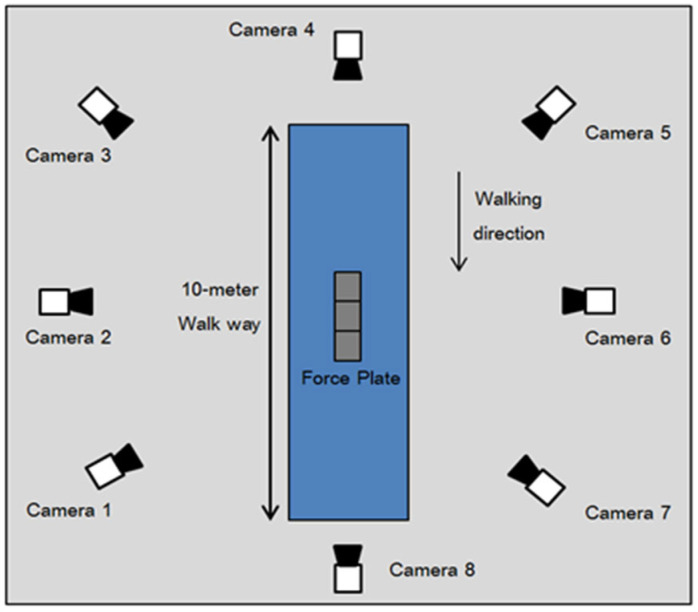
The experimental setup.

Reflective markers were placed on anatomical landmarks according to the Plug-in Gait full-body model, which follows the principles of the Conventional Gait Model and is widely used in clinical gait analysis [19]. A total of 35 reflective markers were attached bilaterally to key bony landmarks. A static calibration trial was first performed in an anatomical standing position with arms relaxed at the sides. Figure 1 and Figure 2 illustrate marker placement and the experimental setup.

Participants completed two warm-up trials, followed by five walking trials at a self-selected comfortable pace along a 10-m walkway, performed barefoot to reduce shoe-related variability. All trials were conducted in a quiet indoor laboratory under consistent lighting and surface conditions. Marker trajectories were filtered and smoothed using the Woltring filter (generalized cross-validatory spline smoothing) within Vicon Nexus software version 1.8.5 to reduce noise while preserving movement fidelity [20]. The generalized cross-validation mode was selected to optimize smoothing based on the inherent characteristics of the kinematic data. This spline smoothing is mathematically equivalent to a dual Butterworth low-pass filter, providing adaptive noise reduction without phase distortion, and is suited for motion capture. The cutoff frequencies for gait kinematic smoothing lie between 6–7 Hz, consistent with the predominant frequency content of human gait signals.

#### 2.2.3. Gait Parameters

Gait events were automatically identified using synchronized vertical ground reaction force (vGRF) data obtained from the force plate and were visually verified based on marker trajectories. Initial contact (heel strike) was defined as the onset of the vertical ground reaction force, whereas toe-off was defined as the instant when the vertical ground reaction force returned to baseline. Kinematic and kinetic data were synchronized within the Vicon system to ensure precise temporal alignment. Each gait cycle was defined from initial contact to the subsequent ipsilateral initial contact. Spatiotemporal and joint kinematic parameters were subsequently calculated using Vicon Polygon™ software version 3.5.2. A minimum of three valid gait cycles per participant were extracted for analysis. A trial was considered valid if complete marker trajectories were captured and clean force plate contact was achieved without overlap from the contralateral limb. The mean values across valid trials were used for statistical analysis. Trials with incomplete marker trajectories or improper force plate contact were excluded from analysis. However, all participants retained at least three valid trials; therefore, the following gait parameters were analyzed:

• Spatiotemporal parameters were calculated using standard biomechanical definitions. Walking velocity was calculated as the horizontal displacement divided by the corresponding time interval (v=d/t). Step length was defined as the anterior–posterior distance between two consecutive initial contacts of opposite limbs, whereas stride length was defined as the distance between two successive initial contacts of the same limb. Step time was calculated as the time interval between consecutive initial contacts of opposite limbs, and stride time as the time interval between successive initial contacts of the same limb. Cadence was calculated as the number of steps per minute ($cadence=\frac{steps}{time}\times 60$). Single support time was defined as the period during which only one limb was in contact with the ground, while double support time was defined as the period during which both limbs were simultaneously in contact with the ground.
• Joint kinematics were computed using three-dimensional segment coordinate systems defined within the Vicon biomechanical model. Joint angles (angular displacement, degrees) were derived from the relative orientation between adjacent segments. Hip, knee, and ankle joint angles were extracted in the sagittal, frontal, and transverse planes at key gait events, including initial contact, mid-stance, toe-off, and mid-swing.

For the KOA group, analyses were performed on the more symptomatic limb as identified through clinical assessment and self-reported pain. In the control group, the dominant limb was analyzed for comparison. All kinematic waveforms were time-normalized to 100% of the gait cycle. Statistical analyses were performed on joint angles extracted at predefined gait events (initial contact, mid-stance, toe-off, and mid-swing).

#### 2.2.4. Statistical Analysis

All statistical analyses were conducted using IBM SPSS Statistics (Version 23.0; IBM Corp., Armonk, NY, USA). The Kolmogorov–Smirnov test was used to assess the normality of data distributions. Homogeneity of variance was evaluated using Levene’s test before conducting independent samples *t*-tests. Independent samples *t*-tests were performed to compare demographic characteristics, WOMAC scores, and gait parameters between the knee osteoarthritis (OA) group and the control group. For categorical variables (i.e., sex), group differences were analyzed using the chi-square test. Statistical significance was set at *p* < 0.05 (two-tailed). Effect sizes (Cohen’s d) were calculated with corresponding 95% confidence intervals to determine the magnitude of between-group differences. Interpretation followed discipline-specific thresholds (0.10 = small, 0.30 = moderate, 0.70 = large), as recommended in recent methodological guidelines [21].

An a priori power analysis was performed using G*Power software (version 3.1) based on previously reported three-dimensional gait biomechanics data in patients with osteoarthritis [7,8]. Assuming an effect size of 0.60, an alpha level of 0.05, and 80% statistical power for a two-tailed independent samples *t*-test, the required sample size was estimated to be 45 participants per group.

## 3. Results

### 3.1. Participant Characteristics

The demographic and clinical characteristics of the participants are presented in Table 1. There were no statistically significant differences between the KOA and control groups with respect to age, sex distribution, height, weight, and body mass index (BMI) (*p* > 0.05). However, participants with knee OA reported significantly higher scores on all domains of the modified Thai Western Ontario and McMaster Universities Osteoarthritis Index (WOMAC), including pain, stiffness, and physical function. These scores indicated the presence of mild to moderate symptom severity in the KOA group, with the total WOMAC score being significantly higher compared to the control group (*p* < 0.01).

### 3.2. Spatiotemporal Parameters

Spatiotemporal gait variables revealed significant differences between the KOA and control groups across all measured domains (Table 2). Participants with KOA demonstrated a notably slower gait velocity and reduced cadence relative to the healthy controls. Additionally, stride length was significantly shorter in the KOA group.

Temporal characteristics also differed significantly between groups. Participants with KOA exhibited longer step time (0.59 ± 0.06 vs. 0.56 ± 0.06 s, *p* = 0.006, d = 0.50) and stride time (1.18 ± 0.13 vs. 1.12 ± 0.12 s, *p* = 0.009, d = 0.48) compared with controls. Moreover, double support time was significantly prolonged in the KOA group (35.80 ± 5.22 vs. 33.11 ± 4.56 s, *p* = 0.009, d = 0.55), while single support time was reduced (32.12 ± 2.80 vs. 33.50 ± 2.39 s, *p* = 0.012, d = −0.53).

### 3.3. Joint Kinematic Parameters

**Figure 3:**
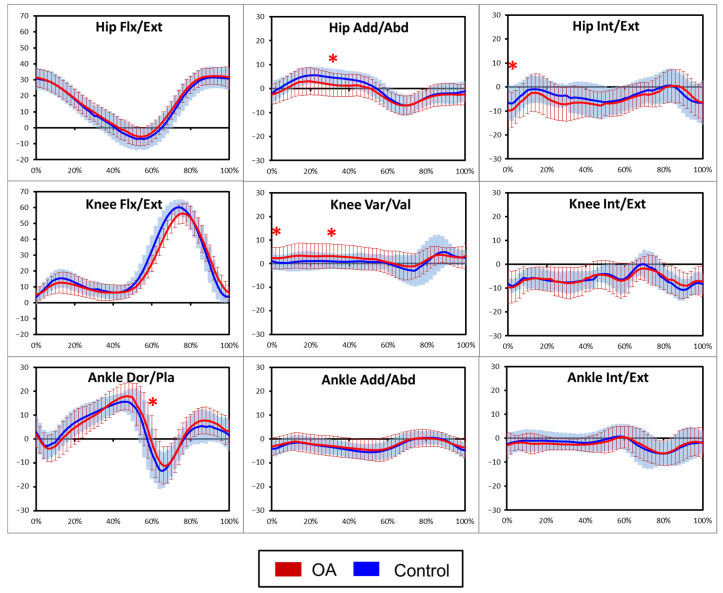
Ensemble average sagittal, coronal and horizontal plane moment waveforms for the hip, knee and ankle during the gait cycle. (Flx: Flexion (+), Ext: Extension (−), Add: Adduction (+), Abd: Abduction (−), Int: Internal rotation (+), Ext: External rotation (−), Var: Varus (+), Val: Valgus (−), Dor: Dorsiflexion (+), Pla: Plantar flexion (−), * indicates statistically significant between-group differences (*p* < 0.05) at the specified gait event.)

Three-dimensional kinematic analysis revealed distinct differences in joint angular displacement across the gait cycle, as illustrated in Figure 3. At initial contact (0% of the gait cycle), individuals with KOA demonstrated significantly greater knee adduction compared to the control group (3.24 ± 4.80 vs. 0.21 ± 3.82 deg, *p* = 0.001, d = 0.70).

At mid-stance (approximately 30% of the gait cycle), the KOA group showed significantly increased hip flexion and reduced hip adduction (2.32 ± 5.06 vs. 5.21 ± 2.84 deg, *p* = 0.002, d = −0.70). Concurrently, the knee remained in greater adduction (i.e., varus alignment) compared to the control group (4.04 ± 5.73 vs. 0.85 ± 4.20 deg, *p* = 0.003, d = 0.62).

Toward toe-off (approximately 60% of the gait cycle), participants with KOA exhibited significantly greater ankle plantarflexion (−5.70 ± 8.07 vs. −10.12 ± 5.93 deg, *p* = 0.004, d = 0.64). Additionally, ankle eversion at initial contact was also more pronounced in the KOA group.

## 4. Discussion

Spatiotemporal and joint kinematic parameters are key determinants of gait alterations in individuals with knee osteoarthritis (KOA) and are often associated with functional limitations and fall risk. In this study, older adults with KOA demonstrated consistent spatiotemporal impairments compared with healthy controls, including reduced gait velocity, cadence, step length, stride length, and single support time, as well as prolonged step time, stride time, and double support time. These findings are consistent with prior studies reporting that individuals with KOA tend to adopt slower and more conservative gait patterns that may reflect protective adaptations to pain, joint loading, and perceived instability [7,8,13].

### 4.1. Spatiotemporal Gait Alterations and Functional Implications

Gait speed is a composite outcome derived from both stride length and cadence. The slower walking speed observed in the KOA group appeared to be associated with concurrent reductions in stride length and cadence, patterns commonly interpreted as cautious gait behavior. Such modifications may indicate altered motor control and neuromuscular coordination in response to joint degeneration, although causal relationships cannot be established in this cross-sectional design. Similar trends have been reported in previous biomechanical studies, where individuals with KOA exhibited lower gait velocity and prolonged double support time—patterns often interpreted as attempts to enhance balance control during ambulation [7,8,9].

The longer double support and shorter single support times observed in this study suggest that individuals with KOA may spend less time in single-limb stance, possibly to reduce mechanical demand on the affected limb. This temporal redistribution may represent a cautious gait pattern that enhances perceived stability, although it may also reflect underlying neuromuscular deficits. Such adaptations could either mitigate or signal elevated fall susceptibility, depending on individual functional capacity. Previous investigations have associated similar gait patterns with quadriceps weakness and increased reliance on proximal musculature for stabilization [8,22].

### 4.2. Multiplanar Joint Kinematics Across Gait Phases

In terms of joint kinematics, our findings provide new insights into multiplanar deviations throughout the gait cycle. At initial contact, the KOA group demonstrated increased knee adduction (varus alignment). The greater knee adduction angle observed at both initial contact and mid-stance is noteworthy, as varus alignment has been associated with altered medial–lateral load distribution in individuals with KOA [14,23]. Although knee adduction moment was not directly measured in the present study, previous research has linked varus alignment with increased medial compartment loading, which is considered an important biomechanical factor in disease progression [24]. Therefore, the present findings should be interpreted cautiously as kinematic indicators rather than direct measures of joint loading.

Mid-stance findings also revealed greater hip flexion and reduced hip adduction in the KOA group, which may reflect altered pelvic control and trunk alignment. These kinematic modifications may indicate proximal compensatory adjustments for impaired knee function, although direct evidence of muscle activation or strength was not obtained. Reduced hip extension reported in previous studies has been linked to gluteal muscle weakness and altered pelvic kinematics, which may contribute to gait asymmetry and reduced propulsion efficiency [22,25].

At toe-off, the KOA group displayed greater ankle plantarflexion compared with controls. This observation may reflect an adaptive ankle strategy to assist forward propulsion when knee extension and quadriceps function are limited. Increased plantarflexion may facilitate push-off and limb advancement when sagittal-plane knee motion is restricted. While previous studies have reported reduced ankle push-off in individuals with more advanced KOA [26], the increased plantarflexion observed in this study may represent a functional adaptation within a milder severity rather than a pathological alteration. Consequently, the clinical significance of this finding should be interpreted cautiously.

Interestingly, no significant between-group differences were observed during the mid-swing phase across joints and movement planes. This phase is characterized by reduced weight-bearing demand and lower joint loading, which may explain the absence of pronounced adaptations. The prior literature similarly suggests that KOA-related gait alterations predominantly occur during stance phases, where mechanical loading and stability demands are greater [9]. The absence of mid-swing differences may therefore indicate that gait adaptations in KOA are primarily load-dependent rather than uniformly distributed across the gait cycle.

### 4.3. Interpretation of Potential Adaptive Gait Patterns and Clinical Implications

Previous studies have demonstrated that gait patterns vary across different severities of knee osteoarthritis (KOA) [7,27,28]. In addition to disease severity, aging itself contributes to joint degeneration and fall-related risk factors that may influence gait performance. The present study specifically focused on older adults with KOA and employed a matched-pairs design to control for age-related influences, while functional status was characterized using WOMAC scores. Collectively, our findings demonstrate characteristic gait alterations in older adults with KOA, including spatiotemporal impairments and multiplanar kinematic deviations. Rather than implying direct causal mechanisms, these alterations may reflect adaptive responses to joint pathology and neuromuscular constraints. The observed patterns highlight the importance of evaluating multiplanar biomechanics when assessing functional mobility and potential fall-related instability in this population.

From a clinical perspective, the identified gait alterations may assist clinicians in recognizing early mobility impairments in individuals with KOA. Cautious spatiotemporal patterns and multiplanar joint deviations during weight-bearing phases may indicate functional instability requiring targeted interventions, including gait retraining, muscle strengthening, and balance-focused rehabilitation. These findings underscore the value of comprehensive three-dimensional gait assessment in supporting individualized rehabilitation planning. However, clinical interpretations should be made cautiously due to the cross-sectional nature of the study.

### 4.4. Limitations

This study has several limitations that should be considered when interpreting the findings. First, participants primarily presented with mild to moderate knee osteoarthritis (KOA), which may limit the generalizability of the results to individuals with more advanced disease. The relatively modest sample size may also reduce the ability to detect subtle kinematic differences, particularly in the transverse plane.

Second, the KOA group lacked detailed clinical stratification. Information regarding radiographic severity (e.g., Kellgren–Lawrence grading), limb dominance, and unilateral versus bilateral involvement was not available, which may influence gait biomechanics and limit disease-specific interpretation. In addition, KOA diagnosis was based on clinical assessment without radiographic confirmation, potentially affecting the clinical homogeneity of the sample.

Third, methodological constraints related to motion analysis should be acknowledged. Although the Plug-in Gait model is widely used in clinical gait assessment, it has recognized limitations in evaluating transverse plane kinematics due to soft tissue artifacts and rotational cross-talk. Furthermore, intra- and inter-rater reliability were not formally assessed, which may affect measurement reproducibility despite the use of standardized protocols and a single experienced examiner.

Fourth, while force plate data were collected, kinetic parameters were not analyzed because the study was specifically designed to focus on spatiotemporal and kinematic outcomes. The absence of kinetic analysis limits comprehensive biomechanical interpretation, particularly regarding joint loading and fall-related mechanisms.

Finally, walking speed differed significantly between groups and was not controlled as a covariate. Because gait kinematics are strongly influenced by walking velocity, some between-group differences may partially reflect speed-related adaptations rather than disease-specific effects.

Future studies incorporating larger samples, detailed clinical classification, longitudinal monitoring, speed-controlled conditions, and integrated kinetic analysis are warranted to provide a more comprehensive understanding of KOA-related gait biomechanics.

## 5. Conclusions

In conclusion, older adults with knee osteoarthritis demonstrated altered spatiotemporal and three-dimensional gait characteristics compared with healthy controls. This study highlights the presence of multiplanar biomechanical alterations across lower-limb joints, particularly during weight-bearing phases. These findings contribute to a more biomechanical understanding of gait adaptations in knee osteoarthritis and may help inform rehabilitation planning aimed at improving functional mobility. However, given the cross-sectional design and potential confounding factors, causal relationships cannot be established.

### Key Points

• This matched-pairs study provides a comparison of three-dimensional gait biomechanics between older adults with knee osteoarthritis and healthy controls.
• Older adults with knee osteoarthritis exhibit distinct spatiotemporal and multiplanar gait alterations involving coordinated joint adaptations at the knee, hip, and ankle, particularly during weight-bearing phases.
• These findings highlight the clinical value of comprehensive three-dimensional gait analysis for functional assessment and individualized rehabilitation planning in this population.

## Figures and Tables

**Table 1:** Characteristics of participants in knee osteoarthritis (KOA) and control groups.

Characteristics	Knee Osteoarthritis (n = 45)	Controls (n = 45)	*p*-Value
Gender (male:female), (n)	10:35	10:35	1.000
Mean age (year)	67.21 ± 6.12	67.49 ± 5.70	0.977
Mean weight (kg)	63.37 ± 10.61	62.06 ± 10.74	0.562
Mean height (cm)	153.35 ± 7.63	153.55 ± 6.60	0.895
Mean BMI (kg/m^2^)	26.96 ± 4.09	26.30 ± 4.06	0.443
Number of medical conditions, median (range)	1 (5)	1 (5)	0.683
Number of medications, median (range)	1 (5)	1 (5)	0.877
Mean modified WOMAC score			
- Pain	13.80 ± 8.71	1.69 ± 3.33	<0.01 *
- Stiffness	3.89 ± 4.11	0.47 ± 1.29	<0.01 *
- Function	35.44 ± 21.81	2.91 ± 6.93	<0.01 *
- Total scores	53.13 ± 31.02	5.07 ± 10.46	<0.01 *

Data reported are mean ± SD. * = Significant difference at *p*-value < 0.01.

**Table 2:** Comparison of spatiotemporal parameters between participants in knee osteoarthritis (KOA) and control group.

Variables	Knee Osteoarthritis (n = 45)	Controls (n = 45)	df	*p*-Value	Effect Size (Cohen’s d) [95% CI]
Velocity (m/s)	0.86 ± 0.14	0.97 ± 0.16	88	0.001 *	−0.73 [−1.16, −0.30]
Cadence (step/min)	102.38 ± 10.72	107.62 ± 10.26	88	0.020 *	−0.50 [−0.92, −0.08]
Step time (s)	0.59 ± 0.06	0.56 ± 0.06	88	0.006 *	0.50 [0.08, 0.9]
Step length (m)	0.50 ± 0.06	0.54 ± 0.07	88	0.071	−0.61 [−1.04, −0.19]
Stride time (s)	1.18 ± 0.13	1.12 ± 0.12	88	0.009 **	0.48 [0.06, 0.90]
Stride length (m)	1.01 ± 0.11	1.08 ± 0.14	88	0.011 *	−0.56 [−0.98, −0.13]
Single support time (% cycle)	32.12 ± 2.80	33.50 ± 2.39	88	0.012 *	−0.53 [−0.95, −0.11]
Double support time (% cycle)	35.80 ± 5.22	33.11 ± 4.56	88	0.009 **	0.55 [0.13, 0.97]

Data reported are mean ± SD. * = Significant difference at *p*-value < 0.05. ** = Significant difference at *p*-value < 0.01.

## Data Availability

The original contributions presented in this study are included in the article. Further inquiries can be directed to the corresponding authors.
